# Amplified fragment length polymorphism and whole genome sequencing: a comparison of methods in the investigation of a nosocomial outbreak with vancomycin resistant enterococci

**DOI:** 10.1186/s13756-019-0604-5

**Published:** 2019-09-23

**Authors:** Victoria A. Janes, Daan W. Notermans, Ingrid J.B. Spijkerman, Caroline E. Visser, Marja E. Jakobs, Robin van Houdt, Rob J.L. Willems, Menno D. de Jong, Constance Schultsz, Sébastien Matamoros

**Affiliations:** 1 Amsterdam UMC, University of Amsterdam, Medical Microbiology, Amsterdam, The Netherlands; 2 Amsterdam UMC, University of Amsterdam, Clinical Genetics, Core Facility Genomics, Amsterdam, The Netherlands; 3 Amsterdam UMC, Vrije Universiteit, Medical Microbiology, Amsterdam, The Netherlands; 4 Department of Medical Microbiology, University Medical Center Utrecht, Utrecht, The Netherlands; 5 Amsterdam UMC, University of Amsterdam, Global Health - Amsterdam Institute for Global Health and Development (AIGHD), Amsterdam, The Netherlands

**Keywords:** WGS, AFLP, Molecular typing, Nosocomial outbreak, VRE

## Abstract

**Background:**

Recognition of nosocomial outbreaks with antimicrobial resistant (AMR) pathogens and appropriate infection prevention measures are essential to limit the consequences of AMR pathogens to patients in hospitals. Because unrelated, but genetically similar AMR pathogens may circulate simultaneously, rapid high-resolution molecular typing methods are needed for outbreak management. We compared amplified fragment length polymorphism (AFLP) and whole genome sequencing (WGS) during a nosocomial outbreak of vancomycin-resistant *Enterococcus faecium* (VRE) that spanned 5 months.

**Methods:**

Hierarchical clustering of AFLP profiles was performed using unweighted pair-grouping and similarity coefficients were calculated with Pearson correlation. For WGS-analysis, core single nucleotide polymorphisms (SNPs) were used to calculate the pairwise distance between isolates, construct a maximum likelihood phylogeny and establish a cut-off for relatedness of epidemiologically linked VRE isolates. SNP-variations in the *vanB* gene cluster were compared to increase the comparative resolution. Technical replicates of 2 isolates were sequenced to determine the number of core-SNPs derived from random sequencing errors.

**Results:**

Of the 721 patients screened for VRE carriage, AFLP assigned isolates of 22 patients to the outbreak cluster. According to WGS, all 22 isolates belonged to ST117 but only 21 grouped in a tight phylogenetic cluster and carried *vanB* resistance gene clusters. Sequencing of technical replicates showed that 4–5 core-SNPs were derived by random sequencing errors. The cut-off for relatedness of epidemiologically linked VRE isolates was established at ≤7 core-SNPs. The discrepant isolate was separated from the index isolate by 61 core-SNPs and the *vanB* gene cluster was absent. In AFLP analysis this discrepant isolate was indistinguishable from the other outbreak isolates, forming a cluster with 92% similarity (cut-off for identical isolates ≥90%). The inclusion of the discrepant isolate in the outbreak resulted in the screening of 250 patients and quarantining of an entire ward.

**Conclusion:**

AFLP was a rapid and affordable screening tool for characterising hospital VRE outbreaks. For in-depth understanding of the outbreak WGS was needed. Compared to AFLP, WGS provided higher resolution typing of VRE isolates with implications for outbreak management.

**Electronic supplementary material:**

The online version of this article (10.1186/s13756-019-0604-5) contains supplementary material, which is available to authorized users.

## Introduction

Antimicrobial resistant (AMR) pathogens commonly cause nosocomial outbreaks. In 2015, AMR pathogens caused an estimated number of 671,689 infections in Europe, of which 63.5% were healthcare associated infections. Of the 33,110 deaths attributable to AMR pathogens, 72.4% were caused by healthcare associated infections [1]. Recognition of nosocomial outbreaks and appropriate infection prevention measures are essential to limit the consequences of AMR pathogens to patients in hospitals.

Because unrelated, but genetically similar AMR pathogens may circulate simultaneously, high-resolution molecular typing methods are needed to determine relatedness of isolates [2]. DNA band-pattern fingerprinting methods, such as amplified fragment length polymorphism (AFLP), accurately confirm relatedness of epidemiologically linked isolates of e.g. *E. coli, P. aeruginosa* and *C. difficile* in hospital outbreak settings [3–5]. Compared to whole genome sequencing (WGS), these methods are still cheaper and faster [3].

However, unrelated strains of bacterial species such as vancomycin resistant *Enterococcus faecium* (VRE) may contain similar genomic regions or mobile genetic elements, thus yielding similar band-patterns hampering their discrimination [5]. Band-patterns may remain identical, while single nucleotide polymorphisms (SNPs) could be present outside the restriction-amplification sites [5, 6]. Thus, highly similar but unrelated isolates may require analysis of the entire genome by whole genome sequencing (WGS) to correctly determine clonality.

The use of WGS for real-time nosocomial pathogen surveillance was proven technically feasible and cost-beneficial [7]. Two retrospective studies demonstrated superiority of WGS over pulse field gel electrophoresis and multi-locus sequence typing to study strain-relatedness of VRE [8, 9]. Comparison of AFLP with a WGS-based essay was done for *E. coli* showing equal performance, but such comparison has not been done for VRE to the best of our knowledge [4].

We describe a retrospective WGS analysis of a nosocomial VRE outbreak for which AFLP was performed as the routine typing method, and investigate the value of WGS for management of VRE outbreaks compared to AFLP.

## Materials and methods

### Hospital setting

This study was performed in a tertiary care university hospital, with approximately 26.000 admissions and 150.000 nursing days per year. The surgery department has three wards and the haematology department one.

### Outbreak management procedures

If *E. faecium* was identified in samples from sterile sites or in pure culture from samples from non-sterile sites, antimicrobial susceptibility testing was performed (see Additional file 5). If the isolate was vancomycin resistant, the patient was placed in contact isolation and contact tracing was initiated among room contacts. Whenever new VRE-carriers were identified, weekly screening was performed amongst ward contacts admitted for at least 3 days on a fixed day of the week. If new VRE carriers were identified in this selected group of ward contacts, contact investigation and isolation was extended to all patients admitted to that ward. An outbreak was defined as temporal and spatial clustering of ≥2 VRE positive patients.

### AFLP

All 26 suspected outbreak isolates were typed by AFLP using EcoRI and MseI as restriction enzymes [10]. AFLP fragment analysis was performed on ABI-3130 (Applied Biosystems). Hierarchical clustering of AFLP types was performed using unweighted pair-grouping (Bionumerics, Applied Maths, Belgium). Similarity coefficients were calculated with Pearson correlation.

### DNA extraction, library preparation and sequencing

DNA was extracted from 1 ml of overnight THY culture, using an enzymatic pre-lysis and the Qiagen DNeasy Blood and Tissue kit (Qiagen, Hilden, Germany) following manufacturer’s instructions for Gram positive bacteria.

The first 10 isolates were sequenced on the Ion Torrent PGM platform (Thermo Fisher Scientific) with a read length of 400 base-pairs (bp). The remaining 19 isolates were sequenced using Illumina MiSeq technology (Illumina, San Diego, CA, USA) with 150 bp paired-end reads (see Additional file 5).

Technical replicates of 2 isolates were sequenced to determine the number of SNPs introduced by sequencing errors. One isolate (S07) was sequenced twice on the Ion Torrent PGM on different runs performed on 2 separate days; the other isolate (H01) on the Ion Torrent PGM and later on the Illumina Miseq platform.

### Bio-informatics sequence analysis

Trimmomatic V0.33 removed poor quality reads [11]. *De-novo* genome assembly was performed with SPAdes3.9 (see Additional file 5) [12]. Contigs < 500 bp were removed. Genome size was calculated using the length of all remaining contigs. Sequence types (ST) were derived using the MLST-tool at https://cge.cbs.dtu.dk/services/MLST [13].

The genomes of 25 VRE isolates collected between 2006 and 2015 in the Netherlands were provided by the University Medical Centre Utrecht for comparison. In addition, five publicly available VRE genomes were randomly chosen from the NCBI database and were added for outlier comparison. Metadata of the included isolates is included in Additional file 4: Table S1.

SNPs were identified using kSNP3.0 [14]. Pairwise SNP differences between isolates were counted using the alignment of the core-SNPs (SNPs at a nucleotide position present in all genomes in the analysed set). The core-SNP alignment was used to build a maximum likelihood tree with RAxML8.2.9 (GTRGAMMA algorithm and rapid bootstrapping) [15]. The diversity estimated by the fraction of core *k-mers* (FCK) in the dataset was 0.634 according to Kchooser [14]. According to Hall, the topological accuracy of maximum likelihood and parsimony algorithms at this level of diversity is comparable, thus maximum likelihood was used for all analyses in this study [16]. iTOL performed tree visualization [17]. In order to check for the presence of recombination events affecting the phylogeny, two different phylogenetic trees (data not shown) were built using Parsnp (http://harvest.readthedocs.io/en/latest/content/parsnp.html/) with the genome of isolate VRE1400294 as reference: one tree using the normal Parsnp algorithm and the second using the recombination filter included in the software (option –x). The topology of both trees was highly similar. In particular the ST117 cluster was entirely similar, with only minor difference in branch length and bootstrap values between both trees [18].

ResFinder3.0 identified and typed vancomycin genes [19]. Geneious11.0.4 aligned and visualised the assembly contigs containing the vancomycin gene cluster [20].

Decision whether isolates belonged to the outbreak cluster was made based on core-SNP distance between genomes, topography in the phylogenetic tree, and SNP variation of the *vanB* gene cluster.

## Results

### Description of the outbreak and AFLP clustering

**Fig. 1:**
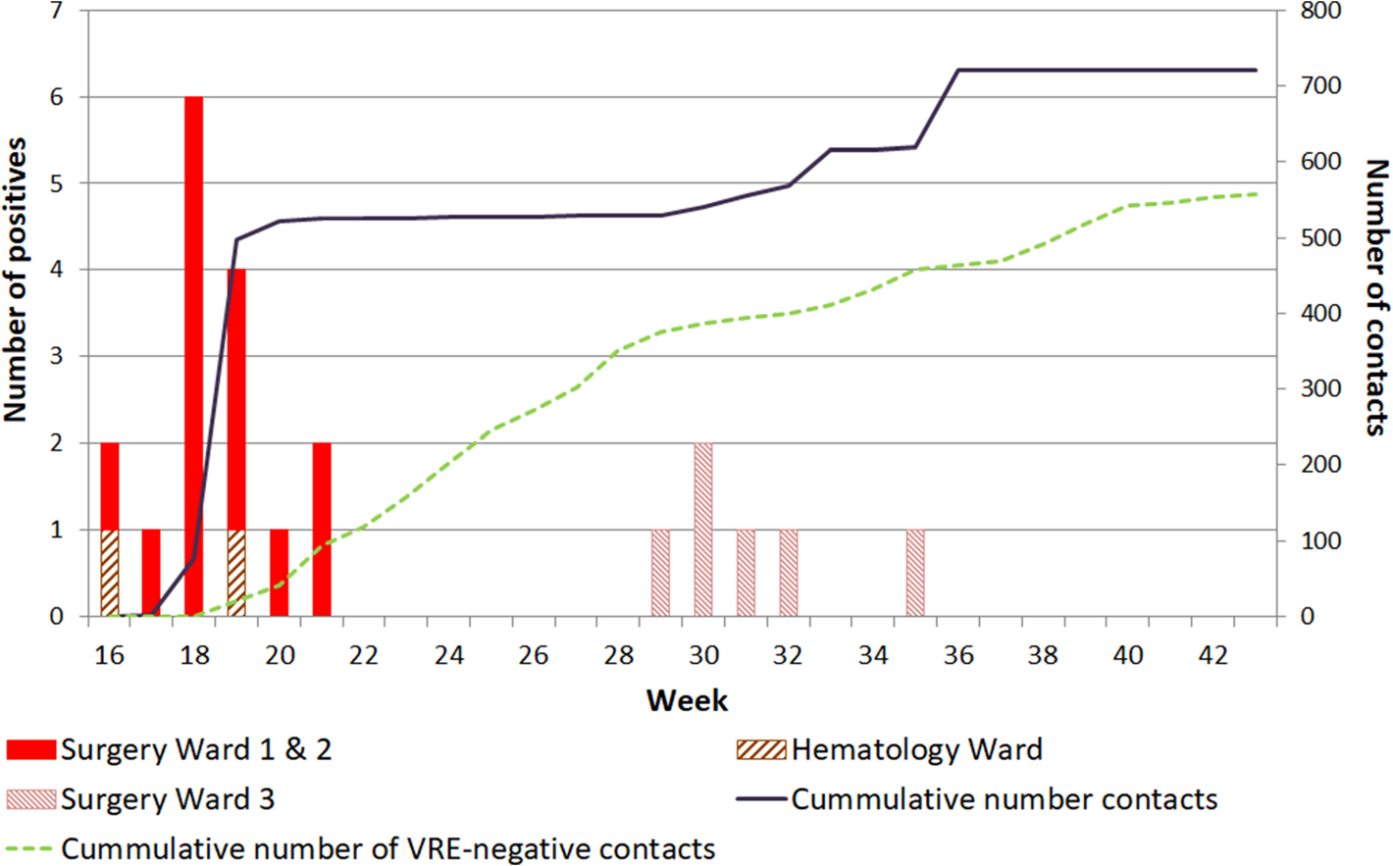
Timeline showing the number of cases and contacts during the VRE outbreak. Bar chart: number of patients that tested positive for a VRE outbreak-type isolate by AFLP (Y-axis, left); solid line: cumulative number of ward and room contacts traced (Y-axis, right); dotted line: room and ward contacts that tested negative for the VRE outbreak-type (Y-axis, right). One hundred thirty-nine patients were traced but lost to follow up. X-axis: week numbers in 2017. The 1st outbreak wave was from week 16–21, the 2nd outbreak wave from week 29–35

The outbreak started with the discovery of VRE in two patients (week 16, 2017). The index patient (S01) was admitted to surgery wards 1 and 2. The second (H01) was admitted to the haematology ward (Fig. 1). These patients had not shared wards, rooms or roommates in the preceding months. Contact tracing was done up to 1 week after discharge, revealing another 14 VRE-positive patients (Fig. 1).

A second outbreak wave was recognized in week 29 with the identification of VRE isolate S15 on surgery ward 3, which had an identical AFLP pattern to the isolates detected in weeks 16–21(Fig. 1). No room or ward contact could be established with the previous VRE-positive patients identified in weeks 16–21. Contact tracing identified 5 more VRE-positive patients, each of whom had been direct room contacts with at least one of the other VRE-positive patients from the 2nd outbreak wave.

In weeks 19 and 35, screening of ward contacts revealed two patients (S07, S20) carrying VRE with AFLP patterns identical to the outbreak strain. This necessitated additional contact tracing and testing of 354 patients (250 for S07, 104 for S20), resulting in the identification of 2 VRE-positive ward contacts of S07 (S10 and S14). Both were part of the outbreak cluster. Subsequent contact tracing did not identify additional cases. Technical replicates of isolate S07 had 96% relative similarity of AFLP band-patterns (cut-off for isolate similarity > 90%, see Additional file 1: Figure S1).

**Fig. 2:**
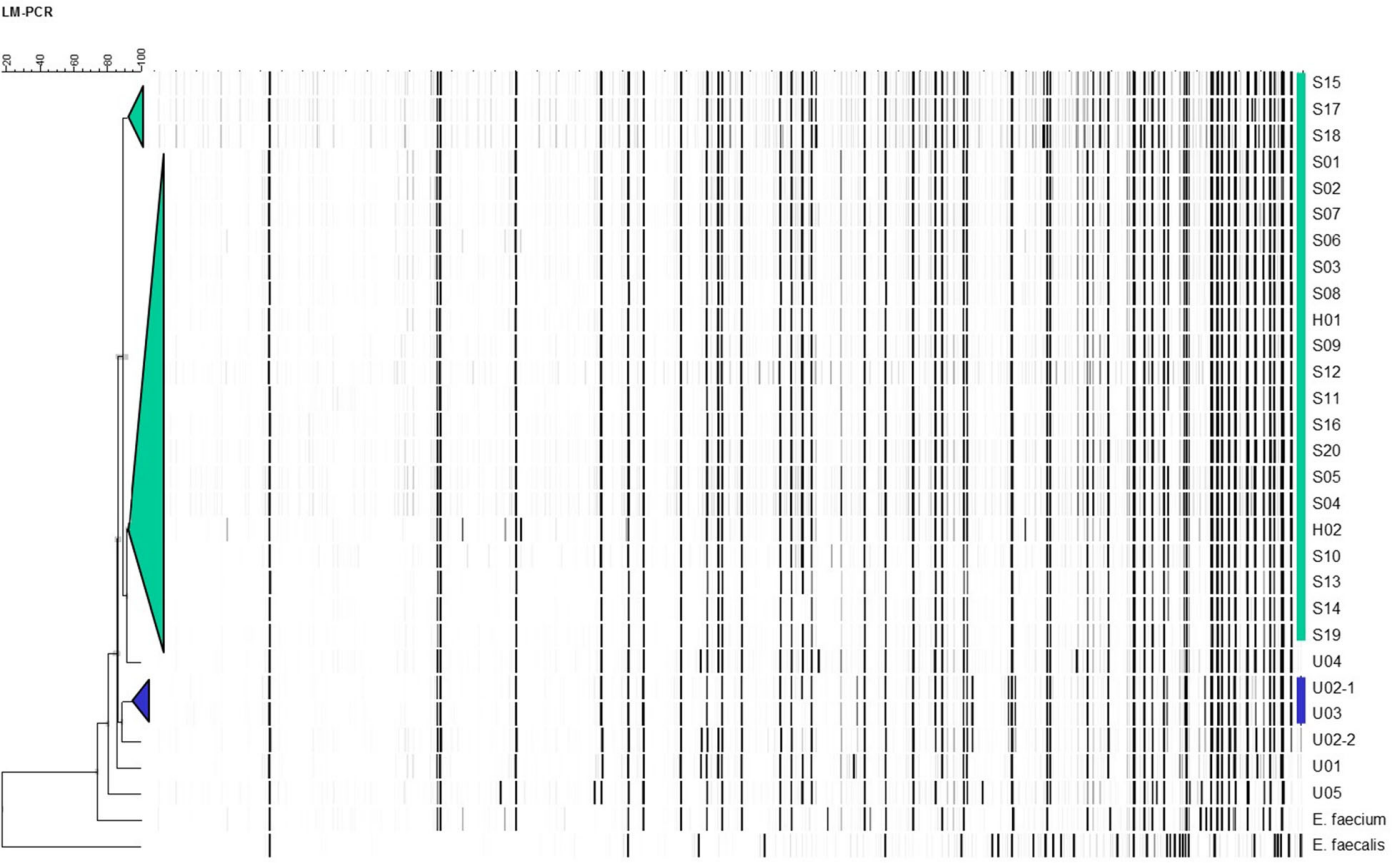
Dendrogram based on AFLP patterns of all VRE strains identified during the outbreak. Unrelated *E. faecium* and *E. faecalis* strains were included in the analysis as outgroup. Hierarchical clustering of AFLP types was performed using unweighted pair-grouping. The cut-off value for identical strains was 90% relative similarity (Bionumerics, Applied Maths, Belgium). For the outbreak strain (in green), the relative similarity was 92%. The relative similarity for S15, S17, and S18 was 89% compared to the outbreak strain. However, this value was deemed to be below 90% due to issues with signal to noise ratios and these three strains were therefore also considered part of the outbreak, based on their AFLP patterns. Unrelated isolates U02–1 and U03 (blue) clustered together with 97% similarity to each other

Summarising, of 721 screened patients, 16 from the first and 6 from the second outbreak wave carried identical VRE isolates according to AFLP (Fig. 2). Vancomycin MICs of all outbreak isolates ranged from 8 to > 16 mg/L by VITEK and from 6 to 16 mg/L by E-test (Additional file 4: Table S1). Among the screened patients, 6 unrelated unique VRE isolates were identified adding to the 560 (78%) that tested negative for the outbreak-type VRE strain. 139 (19%) traced patients were lost to follow-up.

### WGS phylogenetic analysis

**Fig. 3:**
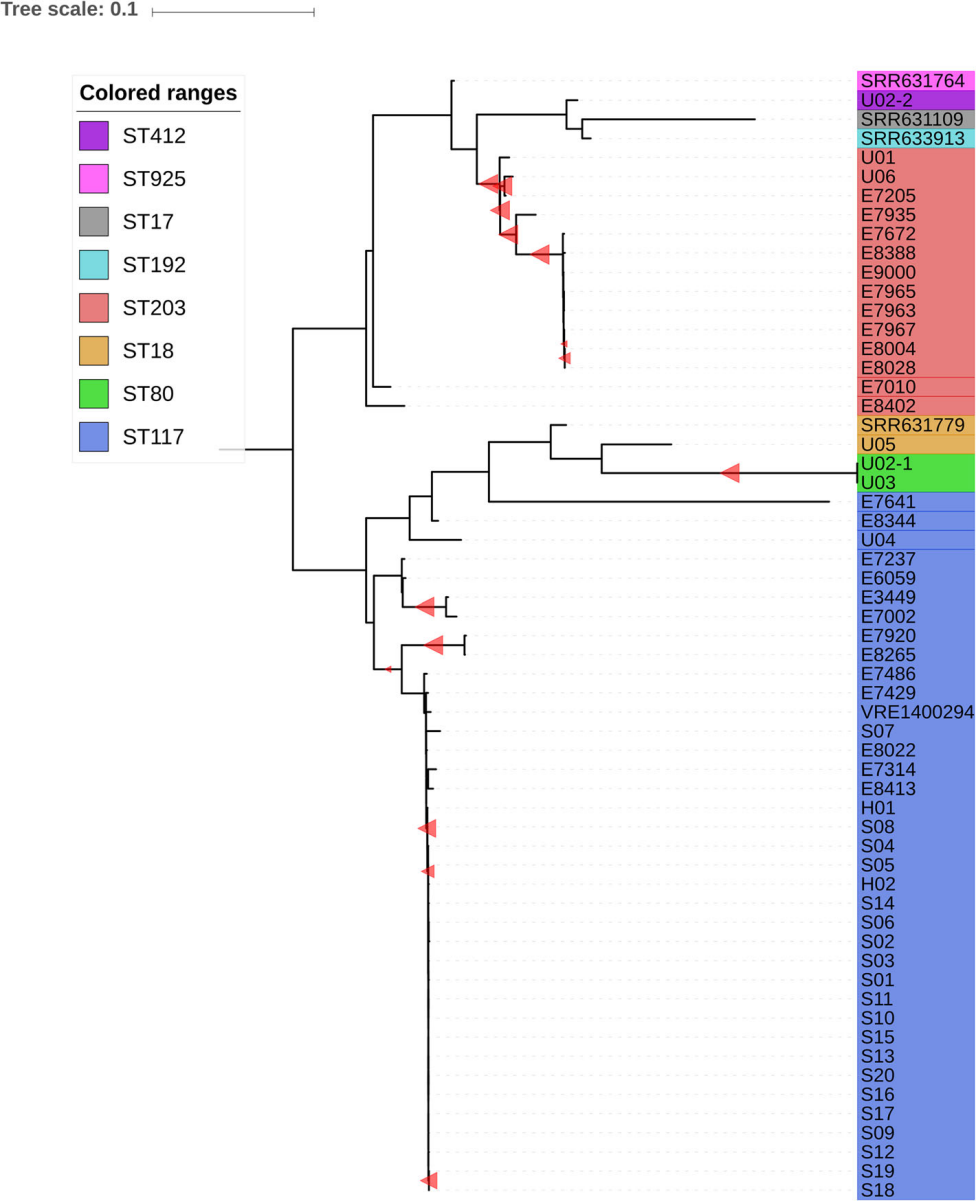
Maximum likelihood tree based on alignment of core-SNPs, mid-point rooted. Included are all outbreak isolates, plus previous isolates collected across the Netherlands (indicated by the letter “E” as the first letter of their name) and unrelated VRE isolates (downloaded from European Nucleotide Association database; indicated by the letters SRR or VRE). SNP count differences with the index isolate (S01), reference free core-SNPs only. Outbreak isolates are numbered and lettered. The letters “S”, “H” and “U” indicate Surgery, Haematology and Unrelated. S, H and U isolates are numbered in chronological order

Of the 721 patients screened for VRE carriage, AFLP assigned the isolates of 22 patients to the outbreak cluster while WGS grouped the isolates of only 21 patients. According to WGS, the number of core-SNPs between each isolate in the outbreak cluster was ≤7. Outbreak isolates belonged to ST117 and carried *vanB*-type resistance gene clusters in their accessory genome. The discrepant result was for isolate S07 (Additional file 4: Table S1). Although WGS identified S07 as ST117, it differed from the index isolate by 61 core-SNPs (Fig. 3, Additional file 4: Table S2). Isolate S07 was re-sequenced, ruling-out technical errors. In addition, no vancomycin resistance gene cluster was identified in either of the sequenced replicates. The initial E-test indicated vancomycin resistance (MIC 8 mg/L), whilst a repeated E-test from frozen stock indicated the isolate was vancomycin susceptible (MIC 0.75 mg/L).

Isolates S08 (surgery ward 1) and H01 (haematology ward) showed 7 core-SNPs differences with the index isolate S01 (Additional file 4: Table S2). H01 and S08 showed 8 core-SNP differences to isolate E8022 recovered in 2014 from a Dutch hospital in a different province, and 24 core-SNPs difference to isolate VRE1400294 recovered from Sweden in 2014 (Additional file 4: Table S2). While the low number of core-SNPs ruled S08 and H01 in the main outbreak cluster, they had only 2 core-SNPs between each other (Fig. 3).

The small branch containing S08 and H01 remained nested within the main outbreak cluster when running the core-SNP analysis on the subset of ST117 isolates. Running the analysis on highly similar isolates, increased the number of *k-mers* included in the analysis, and in turn increased the pairwise SNP differences between these isolates, providing higher resolution for the comparison of closely related isolates. ST117 isolates exhibited 0–79 pairwise core-SNP differences in the analysis comprising all isolates compared to 0–114 core-SNP differences in the ST117-only analysis. In the analysis comprising all isolates, S07 differed by 61 core-SNPs to the index, but in the ST117-only analysis S07 differed 78 core-SNPs to the index. The zoomed-in ST117 phylogeny confirmed the separation between outbreak and non-outbreak isolates and showed that the current outbreak was phylogenetically separated from other Dutch or European circulating clones of ST117, including isolate E8022 (bootstrap value 93%) (Additional file 2: Figure S2).

The *vanB* gene clusters were not part of the core-SNP alignment used to construct the phylogeny. To increase comparative resolution, the *vanB* gene clusters were aligned separately showing that H01 and S08 carried markedly different *vanB* gene clusters from the other Dutch and Swedish isolates E8022 and VRE1400294 (Additional file 3: Figure S3), while their *vanB* gene clusters only differed by 1 SNP from the index isolate S01 (Fig. 3). Additionally, the sequences of H01 and S08 flanking the *vanB* genes were highly similar to all outbreak isolates and dissimilar to E8022 and VRE1400294 (Additional file 3: Figure S3), further supporting the inclusion of this sub-cluster in the main outbreak.

The genomes of all 6 VRE-isolates that were considered unrelated by AFLP were also sequenced. Additionally, an isolate (U06) from a previous VRE outbreak in 2015 in our hospital was included to test for a possible connection between the two outbreaks (Additional file 4: Table S1). In accordance with AFLP, WGS found these isolates to be unrelated to the main outbreak cluster.

Technical replicates of isolate S07 were sequenced on the Ion Torrent PGM on 2 separate runs on 2 separate days, yielding 4 core-SNP differences. Isolate H01 was sequenced on the Ion Torrent PGM and later re-sequenced on the Illumina Miseq platform yielding 5 core-SNP differences, showing a limited number of core-SNPs was introduced by random sequencing errors.

## Discussion

This retrospective study compared AFLP and WGS methods for the epidemiological investigation of a nosocomial VRE outbreak. The two methods differed on the inclusion of 1 out of 22 isolates into the outbreak cluster: AFLP ruled isolate S07 as part of the outbreak, while WGS ruled it out, suggesting a higher discriminative power of WGS compared to AFLP.

A better discrimination of potential outbreak isolates will inevitably have an effect on outbreak management. AFLP ruled isolate S07 into the outbreak which triggered the testing of approximately 250 patients and the quarantining of an entire ward. This required double nursing time per patient and extra cleaning of rooms. Patients isolated for infection control precautions experience more preventable adverse events and have less documented care [21, 22]. Had WGS been used as first-in-line method, this would have been avoided. Interestingly, the contact tracing initiated by the faulty inclusion of S07, identified 2 additional VRE-positive carriers that were part of the outbreak according to WGS (S10 and S14), highlighting the importance of systematic screening of ward contacts.

AFLP is currently still cheaper, faster and less bioinformatics-intensive than WGS. While Nanopore sequencing is also fast and affordable, it is still plagued by sequencing errors necessitating additional short-read sequencing for confident SNP-calling [23]. The typing resolution of AFLP has been shown to be equal to WGS-based methods for typing outbreaks with genetically more stable bacteria than VRE [4, 24, 25]. AFLP groups isolates based on the Msel and Rcol restriction sites while WGS groups isolates based on the entire (core-) genome. Thus, AFLP is likely to include more isolates into the same cluster compared to WGS. This may be preferable in settings where background low-grade transmission is not fully understood. Thus, AFLP is a good first-in-line screening method, particularly to rule-out isolate relatedness, as we demonstrate in this study. If in-depth understanding of transmission routes of the highly similar VRE isolates is required, WGS can still be performed.

Isolate S07 did not carry a *vanB* gene cluster despite its initial vancomycin E-test result indicating resistance. For S07, the first susceptibility test and AFLP typing were done directly after isolation from pure culture, after which the isolate was stored in glycerol at − 80 °C. The frozen stock was used for the repeat AFLP typing, sequencing (twice), and repeat E-test. The repeated identical AFLP patterns of the initial and the frozen isolate preclude an isolate switch (Additional file 1: Figure S1). Thus, the missing *vanB* gene cluster is most likely explained by loss of *vanB* encoding mobile genetic elements during storage, as has been described for *E. faecium* before [26]. Indeed, the repeated E-test from frozen stock indicated vancomycin susceptibility.

Closely related VRE clones are known to circulate in Europe, and horizontal gene transfer of the *vanB* gene cluster has been described [27]. This raised the question as to whether isolates S08 and H01 were part of the outbreak. Although our analysis, based on core-SNP count, phylogenetic clustering and sequence of the *vanB* gene cluster, suggests that they are indeed part of the outbreak, horizontal gene transfer could not be completely ruled-out. Possible low-grade background transmission may go undetected since screening for VRE carriage is limited to high risk patient groups in low-incidence countries such as the Netherlands. Nevertheless, separate introduction from co-circulating similar isolates was less likely, because comparison of the flanking regions of the *vanB* gene cluster showed high similarity between S08, H01 and all other outbreak isolates, while being markedly different from genetically similar but unrelated isolates (Additional file 3: Figure S3).

VRE spread over 10 months was defined as clonal on the basis of ≤20 SNPs difference between isolates based on core-genome MLST [28]. Pinholt et al. found that epidemiologically linked VRE isolates within a single hospital were < 8 core-SNPs apart in 5 months [29], similar to what was described for epidemiologically linked *S. aureus* isolates in a similar time frame (max 11 core-SNPs) [30]. In this light our cut-off of ≤7 core-SNPs for outbreak relatedness of epidemiologically linked isolates was deemed appropriate, further supporting the inclusion of isolates S08 and H01 as part of the outbreak, derived by micro-evolution from the main outbreak cluster.

Since the algorithm used in kSNP is sensitive to the diversity of the analysed dataset, the absolute number of core-SNPs differed when the analysis was performed on the total dataset or only on ST117 isolates [16]. This is a complicating factor in the interpretation of core-SNP phylogenies. However, the number of core-SNPs between outbreak and non-outbreak isolates still differed by almost an order of magnitude in both situations, highlighting the importance of considering the absolute and relative core-SNP counts when defining outbreak clusters. Producing a phylogeny by aligning to a reference genome could be more straightforward, and will allow integral analysis of the *vanB* gene cluster. Finding the appropriate reference genome however may be cumbersome and time consuming, making it less suited towards delivering results in a timely fashion as is required during nosocomial outbreaks.

This study is the first to directly compare AFLP with WGS for typing and management of a nosocomial VRE outbreak. We made use of technical replicates to determine the number of SNPs introduced by sequencing errors, defining a lower detection limit of 5 core-SNPs. We added to the limited body of evidence that a cut-off for relatedness of epidemiologically linked VRE isolates of ≤7 core-SNPs is appropriate for hospital outbreak WGS analysis during 5 months.

## Conclusions

In conclusion, AFLP was a rapid and affordable screening tool for characterising hospital VRE outbreaks, but for full understanding of the outbreak WGS was needed. The increased resolution of WGS compared to AFLP observed in our retrospective study and the fact that sequencing will become faster and cheaper in years to come illustrate the potential of WGS as a primary tool for VRE hospital outbreak control.

## Additional files

Additional file 1: Supplementary methods and scripts. (DOCX 28 kb)

Additional file 2: **Table S1.** All available characteristics of the isolates included in either AFLP, WGS or both analysis methods. NLD = Netherlands, PRT = Portugal, GRC = Greece, DK = Denmark. Phenotypic vancomycin susceptibility testing per typed isolate by VITEK and confirmatory E-test (EUCAST clinical cut-off > 4 mg/L). *The same isolate grown on another plate had an e-test MIC of 8 mg/L. **E-test not done. Same isolate grown on another plate had an e-test MIC of 16 mg/L. **Table S2.** Core-SNP matrix indicating core-SNP differences between each isolate. Boxed are the core-SNP differences between the Index isolate S01 and all other isolates. (XLSX 23 kb)

Additional file 3: **Figure S1.** Dendrogram based on AFLP patterns of the technical replicates of isolate S07. The dendrogram shows two identical band patterns with a relative similarity of 96%. The cut-off value for identical strains was set at 90% relative similarity (Bionumerics, Applied Maths, Belgium). Unrelated *E. faecium* and *E. faecalis* band patterns were added as outlier group comparison. (TIF 1077 kb)

Additional file 4: **Figure S2.** Phylogenetic tree of sequenced ST117 isolates. Included ST117 isolates are: 22 from the current outbreak, 4 recovered from the Netherlands between 2012 and 2015 (E8022, E8413, E7429 and E7486) and 1 isolate recovered from Sweden in 2014 (VRE1400294). The tree shows that H01 and S08 are a small sub-cluster within the outbreak cluster (Bootstrap value = 97%). It also shows how isolate S07, deemed part of the outbreak as per AFLP, is distinctly separated from the outbreak cluster. Isolate VRE1400294 from Sweden showed even closer phylogenetic relatedness to the outbreak cluster than S07. Isolates E8022, E87413, E7429 and E7486 come from four different cities across the Netherlands. The maximum likelihood tree is based on alignment of core-SNPs and is mid-point rooted. Red triangles indicate bootstrap values above 50% (size scaling proportionally from 50 to 100%). (PNG 47 kb)

Additional file 5: **Figure S3.** Alignment of the assembled contigs of isolates containing the *vanB* gene cluster. Aligned are contigs from the outbreak isolates, the unrelated isolates U02–1 and U03 and NCBI isolates KF823968.1, VRE1400294 and E8022. Zoomed in is the *vanB* gene cluster only. SNPs are indicated by black lines. The *vanB* gene clusters of the outbreak isolates have only 8 SNPs difference with the *vanB* gene cluster of non-outbreak isolates U02–1 and U03 (ST80), illustrating the close relatedness of circulating VRE-isolates. Although the sub-cluster isolates H01 and S08 are 1 SNP apart from the index, the regions flanking the *vanB* gene are highly similar to the other outbreak clones and dissimilar to the unrelated isolates VRE1400294 and E8022, supporting inclusion of these isolates in the main outbreak cluster. Alignment was performed with mafft version 3.307 and visualized with Geneious v. 11.04. (PNG 219 kb)

## Data Availability

All data generated or analysed during this study are included in this published article and its supplementary information files. Sequences were deposited in the European Nucleotide Archive (study accession number: PRJEB25629).

## Abbreviations

AFLP: Amplified fragment length polymorphism
AMR: Antimicrobial resistant
MLST: Multilocus sequence typing
SNPs: Single nucleotide polymorphisms
STs: Sequence types
VRE: Vancomycin-resistant *Enterococcus faecium*
WGS: Whole genome sequencing
