# Supplementary material for: Amplified fragment length polymorphism and whole genome sequencing: a comparison of methods in the investigation of a nosocomial outbreak with vancomycin resistant enterococci
Source: Antimicrob Resist Infect Control. 2019 Sep 23;8:153. doi: 10.1186/s13756-019-0604-5 (PMC6757385; doi:10.1186/s13756-019-0604-5)
Supplement: Supplementary file 1 — Supplementary methods and scripts. (DOCX 28 kb) [file 13756_2019_604_MOESM1_ESM.docx]

### Supplementary methods and scripts

**Title: The investigation of a nosocomial outbreak with vancomycin resistant enterococci; a comparison of amplified fragment length polymorphism and whole genome sequencing**

**Authors: Victoria A Janes^1#^, Daan Notermans^1^, Ingrid Spijkerman^1^, Caroline E Visser^1^, Marja E Jakobs^2^, Robin van Houdt^3^, Rob Willems^4^, Menno D de Jong^1^, Constance Schultsz^1,5^, Sébastien Matamoros^1^**

### *Culture conditions and antimicrobial susceptibility testing*

Rectal swabs were cultured overnight in trypticase soy broth with amoxillin (16 mg/L), then plated onto agar containing esculin and bile salts, with vancomycin (6 mg/L) and meropenem (2 mg/L). Black colonies that were identified as *E. faecium* by Malditof-MS (Biotyper Bruker, Bremen, Germany), were further tested for antibiotic resistance by Vitek2 (BioMérieux, Marcy-l’Étoile, France) using EUCAST breakpoints. Resistance (MIC > 4 mg/L) was confirmed using E-test (BioMérieux). VRE isolates were stored at -80°C in 10% glycerol stock.

*DNA extraction, library preparation and sequencing*

Cells were pelleted by centrifuging 1 ml of overnight THY culture at 10.000g for 5 min. After discarding the supernatant, the cell pellet was suspended in 1x TE buffer, enzyme cocktail (achromopeptidase, mutanolysine, lysostaphine (1000:100:3) and lysozyme (1mg/ml) (Sigma-Aldrich, St. Louis, MO, USA) in TE buffer) for an incubation of 45 min at 37 ⁰C. Further DNA extraction and purification steps were performed with the Qiagen DNeasy Blood and Tissue kit (Qiagen, Hilden, Germany) following manufacturer’s instruction for Gram positive bacteria. The Qbit dsDNA HS Assay Kit (ThermoFisher, Waltham, Massachusetts, USA) measured DNA concentration.

The Ion Xpress™ Plus Fragment Library Kit (Thermo Fisher Scientific, Waltham, Massachusetts, USA) was used according to manufacturer’s specifications. Library quantification was performed with the Ion Library TaqMan Quantification Kit (Thermo Fisher Scientific). DNA of the first 10 isolates was sequenced on the Ion Torrent PGM platform, using Ion 318 v2 chips with (Thermo Fisher Scientific) with a read target length of 400 base-pairs (bp). For the remaining isolates, libraries were prepared using the KAPA HTP kit (Roche, Pleasanton, CA, USA) according to manufacturer’s instructions and sequenced using Illumina MiSeq technology (Illumina, San Diego, CA, USA) with 150bp paired-end settings.

*Bio-informatic sequence analysis*

Trimmomatic V0.33 removed poor quality reads(12). *De-novo* genome assembly was performed with SPAdes 3.9(31). Contigs < 500 bp were removed from the genomes to improve overall quality of the assembly. Genome size was calculated using the length of all remaining contigs. Sequence types (ST) were derived by uploading whole genomes to the web-based MLST-tool at https://cge.cbs.dtu.dk/services/MLST(14).

The genomes of 25 VRE isolates collected between 2006 and 2015 were provided by the University Medical Centre Utrecht, Utrecht, Netherlands for comparison. Five randomly chosen additional publicly available VRE genomes were added to the analysis for comparison. A complete list of included isolates and their characteristics is included in Supplementary Table 1.

Single nucleotide polymorphisms (SNPs) between the bacterial genomes were identified using kSNP3.0(15). Briefly, kSNP 3.0 identifies SNPs in a set of microbial genomes by comparing k-mers of a pre-selected length (in this study: 19bp) without alignment to a reference genome. The pairwise SNP differences between isolates were counted using the core-SNPs (SNPs at a location present in all genomes in the analysed set) alignment output.

The diversity estimated by the fraction of core *k-mers* (FCK) in the dataset was 0.634 according to Kchooser(14). According to Hall, the topological accuracy of maximum likelihood and parsimony algorithms at this level of diversity is comparable, thus maximum likelihood was used for all analyses in this study(16). iTOL performed tree visualization(17). In order to check for the presence of recombination events affecting the phylogeny, two different phylogenetic trees (data not shown) were built using Parsnp (<http://harvest.readthedocs.io/en/latest/content/parsnp.html/>) with the genome of isolate VRE1400294 as reference: one tree using the normal Parsnp algorithm and the second using the recombination filter included in the software (option –x). The topology of both trees was highly similar. In particular the ST117 cluster was entirely similar, with only minor difference in branch length and bootstrap values between both trees(18).

Vancomycin genes were identified and typed using ResFinder3.0(20). Vancomycin gene cluster containing contigs were aligned and visualized with Geneious11.0.4(21). Decision whether isolates belonged to the outbreak cluster was made based on the combination of core-SNP distance between genomes, topography of the phylogenetic tree and SNP variation of the *vanB* gene cluster. Costs and Turnaround time (TAT) were monitored for AFLP and WGS.

**Supplementary scripts**

#########################

# Reads quality trimming with trimmomatic 0.33

#########################

# IonTorrent

java -jar trimmomatic-0.33.jar SE S1.fastq S1_trimmed.fastq SLIDINGWINDOW:4:15 LEADING:3 TRAILING:3 MINLEN:50

# Illumina paired-end reads

java -jar trimmomatic-0.33.jar PE S15_R1.fastq S1_R2.fastq S15_R1_P_trimmed.fastq S15_R1_UP_trimmed.fastq S15_R2_P_trimmed.fastq S15_R2_UP_trimmed.fastq SLIDINGWINDOW:4:15 LEADING:3 TRAILING:3 MINLEN:36

#########################

# Assembly with Spades 3.9.0

#########################

# IonTorrent

spades.py --iontorrent -k 21,33,55 --careful -s S1_trimmed.fastq -o spades_output_S1

# Illumina paired-end reads

spades.py -1 S15_R1_P_trimmed.fastq -2 S15_R2_P_trimmed.fastq -o spades_output_S15 --careful

#########################

# Core SNP detection with kSNP3

#########################

kSNP3 -in vre_isolates_path_list.txt -outdir vre_isolates_ksnp3_out -k 19 -core

#########################

#Maximum likelihood tree with RAxML

#########################

raxmlHPC-PTHREADS-SSE3 -T 16 -m GTRCAT -p 31766040 -f a -x 7417 -N autoMRE -s vre_isolates_ksnp3_out_core_SNPs_matrix.fasta -n vre_isolates_ksnp3_out_core_SNPs_matrix -w /vre_isolates_core_SNPs_raxml_out/
