# Supplementary figures and images for: Amplified fragment length polymorphism and whole genome sequencing: a comparison of methods in the investigation of a nosocomial outbreak with vancomycin resistant enterococci
Source: Antimicrob Resist Infect Control. 2019 Sep 23;8:153. doi: 10.1186/s13756-019-0604-5 (PMC6757385; doi:10.1186/s13756-019-0604-5)

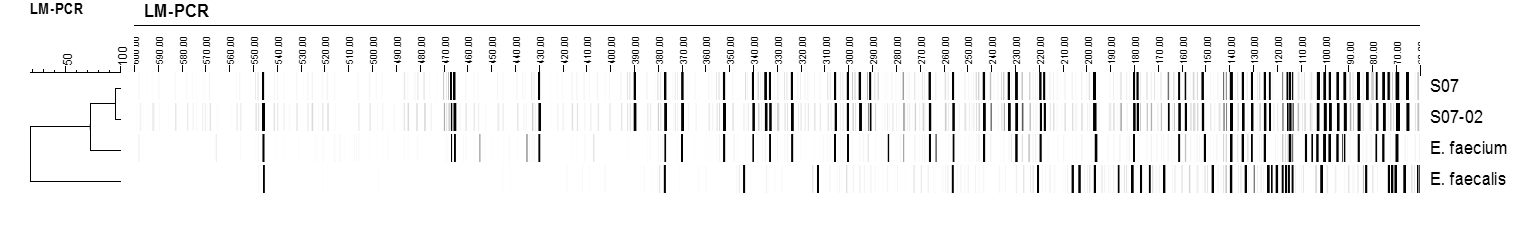

Supplement: Supplementary file 3 — Figure S1. Dendrogram based on AFLP patterns of the technical replicates of isolate S07. The dendrogram shows two identical band patterns with a relative similarity of 96%. The cut-off value for identical strains was set at 90% relative similarity (Bionumerics, Applied Maths, Belgium). Unrelated E. faecium and E. faecalis band patterns were added as outlier group comparison. (TIF 1077 kb) [file 13756_2019_604_MOESM3_ESM.tif]

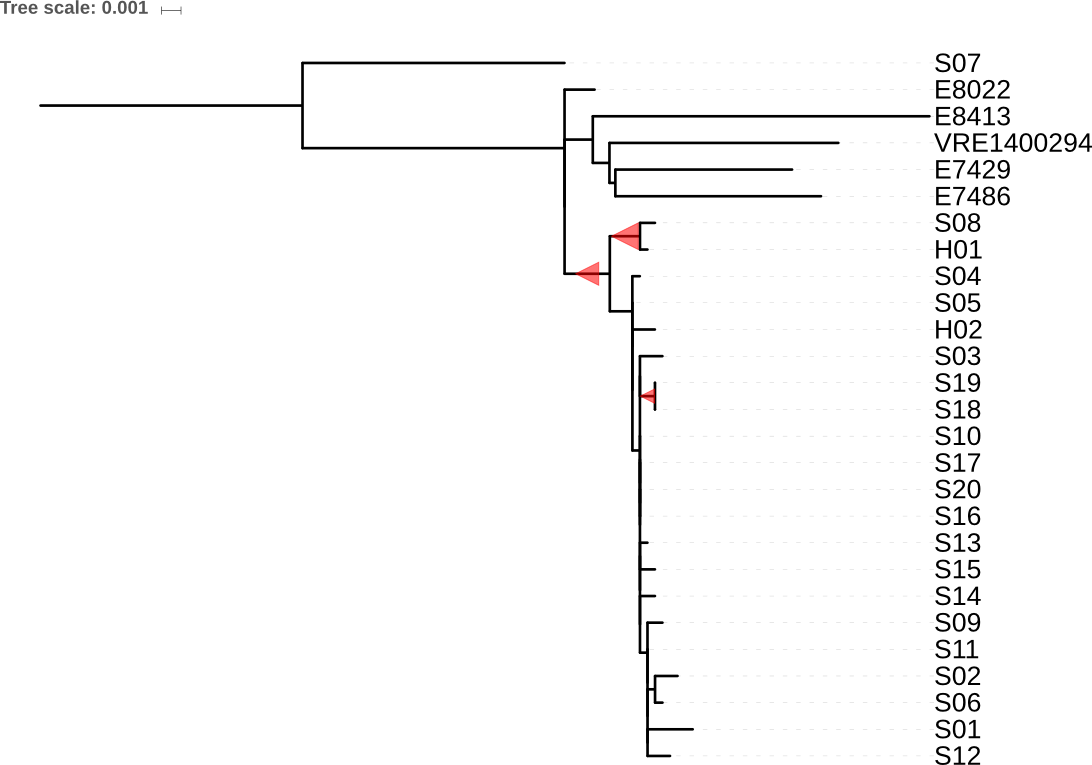

Supplement: Supplementary file 4 — Figure S2. Phylogenetic tree of sequenced ST117 isolates. Included ST117 isolates are: 22 from the current outbreak, 4 recovered from the Netherlands between 2012 and 2015 (E8022, E8413, E7429 and E7486) and 1 isolate recovered from Sweden in 2014 (VRE1400294). The tree shows that H01 and S08 are a small sub-cluster within the outbreak cluster (Bootstrap value = 97%). It also shows how isolate S07, deemed part of the outbreak as per AFLP, is distinctly separated from the outbreak cluster. Isolate VRE1400294 from Sweden showed even closer phylogenetic relatedness to the outbreak cluster than S07. Isolates E8022, E87413, E7429 and E7486 come from four different cities across the Netherlands. The maximum likelihood tree is based on alignment of core-SNPs and is mid-point rooted. Red triangles indicate bootstrap values above 50% (size scaling proportionally from 50 to 100%). (PNG 47 kb) [file 13756_2019_604_MOESM4_ESM.png]

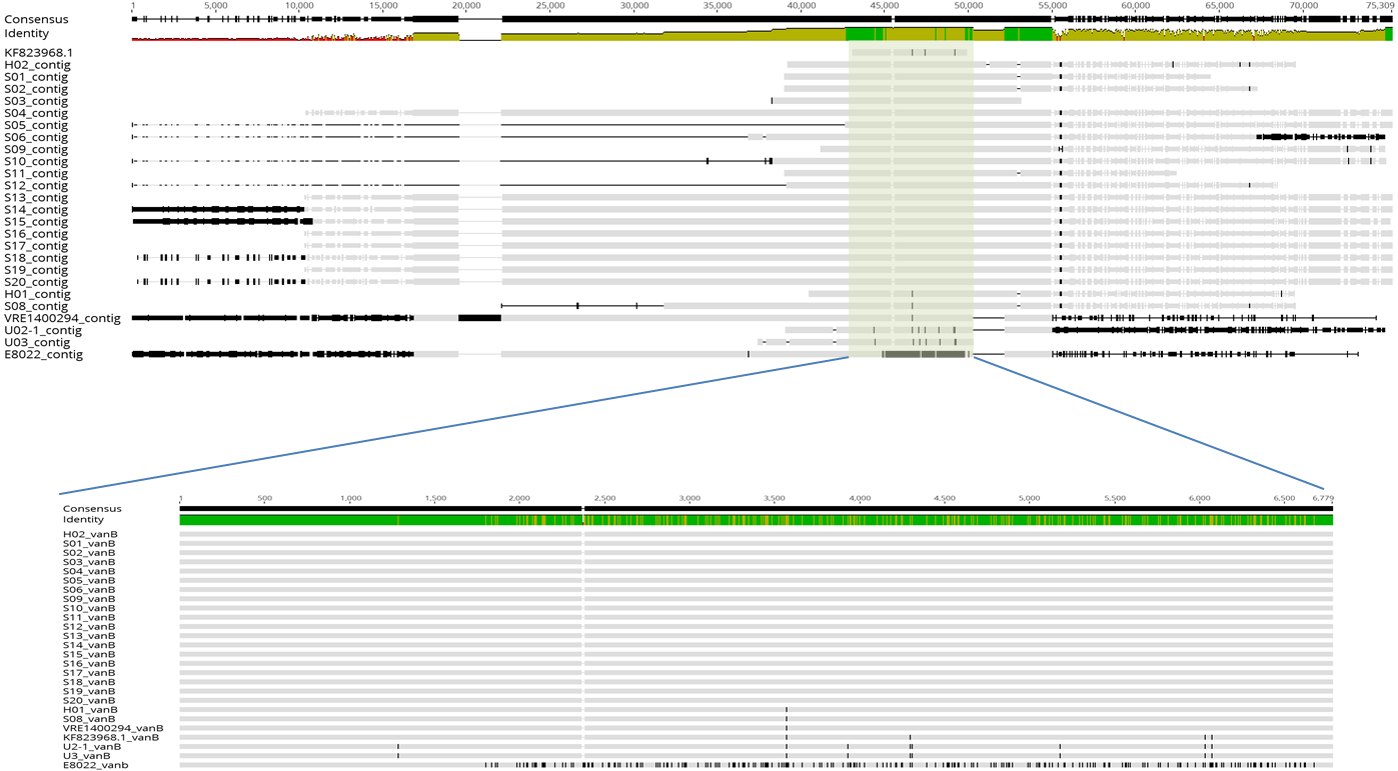

Supplement: Supplementary file 5 — Figure S3. Alignment of the assembled contigs of isolates containing the vanB gene cluster. Aligned are contigs from the outbreak isolates, the unrelated isolates U02–1 and U03 and NCBI isolates KF823968.1, VRE1400294 and E8022. Zoomed in is the vanB gene cluster only. SNPs are indicated by black lines. The vanB gene clusters of the outbreak isolates have only 8 SNPs difference with the vanB gene cluster of non-outbreak isolates U02–1 and U03 (ST80), illustrating the close relatedness of circulating VRE-isolates. Although the sub-cluster isolates H01 and S08 are 1 SNP apart from the index, the regions flanking the vanB gene are highly similar to the other outbreak clones and dissimilar to the unrelated isolates VRE1400294 and E8022, supporting inclusion of these isolates in the main outbreak cluster. Alignment was performed with mafft version 3.307 and visualized with Geneious v. 11.04. (PNG 219 kb) [file 13756_2019_604_MOESM5_ESM.png]
